# Seroprevalence and genetic diversity of feline immunodeficiency virus in outdoor cats in France

**DOI:** 10.1186/s13567-025-01672-z

**Published:** 2025-12-04

**Authors:** Pierre Bessière, Brandon Hayes, Maxime Fusade-Boyer, Aurélie Sécula, Gabin Rous, Jessie Brun, Amélie Marchand, Guillaume Croville, Marie-Christine Cadiergues, Jean-Luc Guérin

**Affiliations:** 1 Univ Toulouse, ENVT, INRAE, IHAP, Toulouse, France; 2 Univ Toulouse, ENVT, Toulouse, France

**Keywords:** Virology, FIV, feline, seroprevalence

## Abstract

Feline immunodeficiency virus (FIV) is a retrovirus that causes lifelong infections in cats and may lead to immune dysfunction. Despite its importance for feline health, there is limited FIV data from France. This study investigated samples collected from stray and owned cats with outdoor access across France between December 2023 and January 2025 to estimate FIV seroprevalence, identify seropositivity predictors and analyse the genetic diversity of circulating strains. Serological screening was performed using a commercial ELISA. Polymerase chain reaction (PCR) was conducted on ELISA-positive sera, with selected samples analysed by Sanger sequencing for phylogenetic inference. One sample underwent metagenomic shotgun sequencing using Oxford Nanopore technology. The national seroprevalence, estimated using a Bayesian hierarchical model, was 16% (95% credible interval: 8.4–20%) overall, then 31% (21–42%) among intact male cats, 18% (CrI: 10.6–25.2%) among neutered male cats and 8.4% (CrI: 1.8–14%) among female cats. Outdoor exposure, sex and neuter status were strong predictors of seropositivity. Among strays, predicted probability of seropositivity exceeded 50% by 5 years of age. All sequenced viruses were classified as subtype A. However, the phylogenetic analysis revealed notable genetic variability, indicating at least two independent introductions of FIV into France. While related to other European strains, several isolates appeared to share distinct ancestral lineages. The metagenomic dataset yielded approximately 100,000 FIV reads among 2 million total reads, enabling full genome recovery. These findings highlight the ongoing circulation of FIV in France and provide valuable data for veterinary practitioners and future surveillance efforts in Europe.

**Supplementary Information:**

The online version contains supplementary material available at 10.1186/s13567-025-01672-z.

## Introduction

Feline immunodeficiency virus (FIV) is an enveloped retrovirus that belongs to the *Retroviridae* family, *Orthoretrovirinae* subfamily and *Lentivirus* genus. It is characterized by a spherical morphology and a positive-sense, single-stranded RNA genome. First isolated in 1986 in California, FIV is believed to have originated several million years ago in wild African felids, where related lentiviruses are enzootic [1, 2].

As with all RNA viruses, FIV forms a viral quasispecies in vivo [3], although its within-host diversity is relatively low compared with human immunodeficiency virus (HIV) [4]. FIV is classified into seven subtypes based on genetic variation within the *env* gene [5]. Although some geographic patterns of subtype distribution have been described, there is currently no convincing evidence of subtype-specific differences in pathogenicity [6]. The virus is globally distributed, with seroprevalence estimates varying widely by region [7]. In Europe, reported prevalence rates reach up to 35%. In France, data remain sparse; the most recent national study, published in 2011, reported a prevalence of 16.1% [8].

FIV is primarily transmitted through deep bite wounds, most often during territorial fights among outdoor or free-roaming male cats. Once infected, cats remain lifelong carriers [9]. Seroconversion typically occurs within 60 days post-exposure, and anti-FIV IgG antibodies become detectable as early as 2–4 weeks after infection in experimentally infected cats [10]. The infection is classically diagnosed by the detection of antibodies using enzyme-linked immunosorbent assay (ELISA) or point-of-care (POC) tests [11].

Given the limited and outdated data available for France, the present study aimed to (i) assess the current seroprevalence of FIV among adult outdoor cats across the country using a commercial ELISA test, (ii) identify individual and environmental predictors for seropositivity and (iii) assess FIV genetic diversity. To our knowledge, this is the first large-scale investigation of FIV in French cats in over a decade based on robust serological methods.

## Materials and methods

### Ethics

Both approval from the Science et Santé Animale n°115 Ethics Committee of the National Veterinary School of Toulouse (SSA_2023_021) and written owner consent were obtained in each case prior to the study.

### Sample collection

The sera used were those from a previous study, in which 728 cats with outdoor access (642 domestic and 86 stray) were sampled between December 2023 and January 2025 in 44 veterinary clinics across mainland France [12]. A total of 19 cats had antibodies against H5Nx avian influenza viruses. A total of 329 were female (113 intact, 216 neutered), 371 were male (115 intact, 256 neutered) and sex was unknown for 28 animals. The average age was 6.6 years. These animals were presented to their veterinarian for various reasons, ranging from routine check-ups to consultations for different medical issues. The sera were frozen at –20 °C until transport under positive cold conditions. Upon reception, they were aliquoted and stored at –80 °C.

### ELISA

Serological status of the cats was assessed using a commercial FIV enzyme-linked immunosorbent immunosorbent assay (ELISA) in accordance with the manufacturer’s instruction (VetLine FIV ELISA, NovaTc Immunodiagnostica GmbH). According to the manufacturer, sensitivity and specificity were 0.955 and 0.963, respectively.

### RNA extraction

Viral RNA was extracted from 50 μL of serum using the magnetic bead-based ID Gene Mag Fast Extraction Kit and an IDEAL-96 automated platform, according to the manufacturer’s instructions (Innovative Diagnostics, Grabel, France).

### RT-qPCR

FIV nucleic acid load was determined by RT-qPCR performed in 96-well plates in a final volume of 20 µL using a LightCycler 96 system (Roche, Mannheim, Germany). Mixes were prepared according to the manufacturer’s instructions (iTaq SYBR green one-step, Bio-Rad) with 2 μL of RNA and a final concentration of 0.5 μM of each primer. For phylogenetic analysis, a 550 bp fragment including the V3–V5 region of the *env* gene was amplified by nested PCR as previously described [13], using Qiagen OneStep RT-PCR kit (Qiagen, Canada) and GoTaq Green Master Mix (Promega, USA) for the first and second amplification round respectively, according to the manufacturer’s instructions. PCR cycling conditions were: 5 cycles of 94 °C/1 min, 50 °C/1 min, 72 °C/2 min; 72 °C/5 min; 30 cycles of 94 °C/15 s, 55 °C/45 s, 72 °C/2 min; 72 °C/5 min. In the second round of PCR, the extension period during the 30 cycles was 72 °C for 1 min. Primer sequences are available in Table 1.

**Table 1:** **Primers used for PCR**

	Target	Primers sequences (5’ to 3’)	References
Screening	*gag*	F: TGGAGCACCACAATATGTAGC R: CTAATGCCTCAAGATACCATGC	[42]
Phylogeny	*env* (round 1)	F: GCTCAGGTAGTATGGAGACT R: ACTTCATCATTCCTCCTCTT	[13]
	*env* (round 2)	F: ATACCAAAATGTGGATGGTG R: CAAGACCAATTTCCAGCAAT	

### Phylogenetic analysis

Phylogenetic analysis was performed on the V3–V5 region of the *env* gene, which allows subtype classification and is the gene’s most variable region. A total of 15 samples were selected for phylogenetic analysis so that each of the 16 departments (French administrative units) included in the study would be represented (one department had to be excluded, as we were unable to successfully sequence any sample from it). Sequences of all samples were compared with the most related sequences available in Genbank. An alignment was performed with MAFFT version 7. After identification of the most suitable model for the analysis using IQ-Tree software, version 1.6.12, maximum likelihood phylogenetic trees were generated using the General Time Reversible model (GTR) with empirical base frequencies (F), and a free rate model of rate heterogeneity across sites with five categories (R5), with 10,000 replicates using ultrafast bootstraps. Phylogenetic trees were then visualized by using FigTree version 1.4.2.

### Statistical analysis

Serological test data was analysed across 33 departments in metropolitan France. Each observation included departments of sample origin, age, sex, neuter status, estimated time spent outdoors (binned to less than an hour, between 1 and 12 h, or more than 12 h), and whether the cat was owned or a stray. Missing values for age (*n* = 35), sex (*n* = 28), neuter status (*n* = 28) and time spent outdoors (*n* = 32) were imputed via predictive mean matching (age), logistic regression (sex, neuter status) or polytomous regression (time outdoors). Descriptive characteristics of the final sample population are available in Additional file 1. To resolve collinearity between stray status and time spent outdoors, a composite variable of outdoor exposure was created that collapsed the combinations into ordered and mutually exclusive categories reflecting cumulative FIV exposure risk: low exposure (owned cats never outdoors or outdoors for less than 1 h per day), moderate exposure (owned cats reported outside for more than 1 h but less than 12 h), high exposure (owned cats outside for more than 12 h) and stray cats (presumed always outside). Sex and neuter status were combined into three categories that reflect known risks with FIV: intact males, neutered males and females.

National prevalence was computed using a Bayesian hierarchical model designed to account for diagnostic classification error [14], incorporating known variability in both sensitivity (84.53–99.44%) and specificity (89.43–99.22%) of the VetLine Feline Immunodeficiency Virus (FIV) ELISA [15]. Using a binomial likelihood, the number of observed test-positive cats was modelled as a function of the latent true prevalence and the ELISA’s diagnostic characteristics. Sensitivity and specificity were assigned beta-distributed priors and parameterized from the contingency table of manufacturer validation data (42 true positives, 2 false negatives, 3 false positives and 77 true negatives). Posterior sampling was performed with JAGS, interfaced through R via the RJAGS package, using 10,000 MCMC iterations following a 1000 iteration burn-in [16–18]. Chain convergence and model adequacy was assessed via visual inspection of trace plots. Prevalence estimates were computed overall, within the stray and owned populations, and for male intact, male neutered and female cat populations, with differences between groups evaluated through the comparison of sample posterior means. Bayesian results were then compared to Rogan–Gladen estimates, using manufacturer sensitivity and specificity of 0.955 and 0.963, respectively.

The association between FIV seropositivity and the exposure predictors age, sex-neuter status and outdoor exposure was evaluated through four approaches: a logistic regression model (GLM) with fixed effects, a generalized linear mixed model (GLMM) with department-of-origin as a random intercept, a spatial model using a Bessag–York–Mollié (BYM2) structured random effect for department adjacency and implemented via integrated nested Laplace approximation (INLA) and a fully Bayesian GLMM with weakly informative priors (BRM). Multiple modelling frameworks were employed due to the spatial nature of the data, and to determine whether spatial autocorrelation or department-level clustering (i.e. hierarchical structure) influenced our estimates. Here, the GLMM and BRM are non-spatial hierarchical models that include department-level random intercepts to account for clustering of cats within departments. Conversely, the INLA model explicitly incorporates adjacency-based structured effects (to account for spatial autocorrelation) along with an unstructured component to capture residual heterogeneity across departments. For both the BRM and GLMM, department status was modelled as a non-spatial group-level random effect. Model outputs consisted of odds estimates and 95% confidence intervals (for GLM/GLMM) or credible intervals (for INLA/BRM). Model performance was assessed through Akaike Information Criterion (AIC) for frequentist models (GLM and GLMM) and Watanabe–Akaike Information Criterion (WAIC) for Bayesian models (BRM and INLA). Models were compared directly within a given framework by associated quantitative metrics.

Prior to calibration, given the high proportion of intact male cats observed among the stray cohort, multicollinearity was assessed to account for association between sex and outdoors exposure through generalized variance inflation factors (GVIF). Only minimal association was noted (all GVIF < 1.25), and both variables were retained in the models.

To evaluate the association between age and FIV seropositivity among strays, the best-performing model framework was fit using age as a continuous predictor, and predicted probabilities of FIV seropositivity were then computed.

Statistical analysis was performed in R using the mice, lme4, INLA and brms packages, along with the tidyverse suite for data manipulation and visualization [19–23].

### Metagenomic

#### Viral RNA concentration

A 40 µL volume of viral RNA extract was purified and concentrated using the RNA Clean and Concentrator-5 kit (Zymo Research, USA), following the manufacturer’s instructions. Final elution was performed in 10 µL of RNase-free water.

#### DNase treatment

To eliminate genomic DNA contamination, an enzymatic treatment was carried out using the ezDNase kit (Thermo Fisher Scientific, USA). The final 10 µL reaction mixture consisted of 8 µL of concentrated RNA, 1 µL of ezDNase and 1 µL of reaction buffer. The mix was incubated at 37 °C for 2 min. Enzyme inactivation was achieved by adding 1 µL of 100 mM DTT, and by incubation at 55 °C for 5 min. The sample was then kept on ice until further processing.

#### cDNA synthesis, PCR amplification and purification

cDNA synthesis and PCR amplification were performed according to the SMART-9N protocol [24]. DTT was omitted from the reverse transcription mix, as it had already been added during the DNase inactivation step. The entire 11 µL of DNase-treated RNA was used as a template. PCR amplification was performed in a final reaction volume of 50 µL, using the Q5 High-Fidelity Master Mix (New England Biolabs), with all reagent volumes doubled relative to the original SMART-9N protocol. PCR products (50 µL) were purified and concentrated using AMPure XP beads (Beckman Coulter) at a bead-to-sample volume ratio of 0.5:1. Final elution was performed in 15 µL of RNase-free water.

#### Sequencing library preparation and loading

Purified PCR products were quantified using the TapeStation system (Agilent Technologies) with the D5000 kit. A total of 50 ng were used for library preparation, using the Rapid Barcoding Kit 24 V14 (Oxford Nanopore Technologies), then loaded onto a PromethION FLO-PRO114M flowcell for sequencing on the PromethION 2 Solo, using MinKNOW v24.11.10.

#### Basecalling and quality filtering

Basecalling was performed in real time using Dorado v7.6.8, with a minimum quality score (Q score) threshold of 10 applied to retain high-confidence reads.

#### Bioinformatics analysis

The sample analysed corresponded to barcode 2 of the multiplexed library. Raw FASTQ files were filtered using fastplong v0.2.2 [25], applying a minimum read length threshold of 500 bp, and 30 bp were trimmed from both the 5′ and 3′ ends of each read to remove sequences introduced during the SMART-9N protocol. Taxonomic classification was performed with Kraken2 v2.1.2 [26], using a viral RefSeq database. Reads assigned to taxon 11,673, corresponding to FIV, were extracted using KrakenTools (commit d4a2fbe) [27], then aligned to a reference genome (NCBI Reference Sequence: NC_001482.1) using Minimap2 v2.28 [28] and Samtools v1.19 [29]. A consensus sequence was generated from this alignment using iVar v1.4.2 [30], with a minimum base quality threshold (Phred score) of 20 applied for base calling. Finally, to validate the assembly and assess genome coverage, the filtered reads were realigned to the consensus sequence using Minimap2 and Samtools. The resulting alignments were then visualized with IGV v2.17.4.

## Results

### Sampling effort

**Figure 1:**
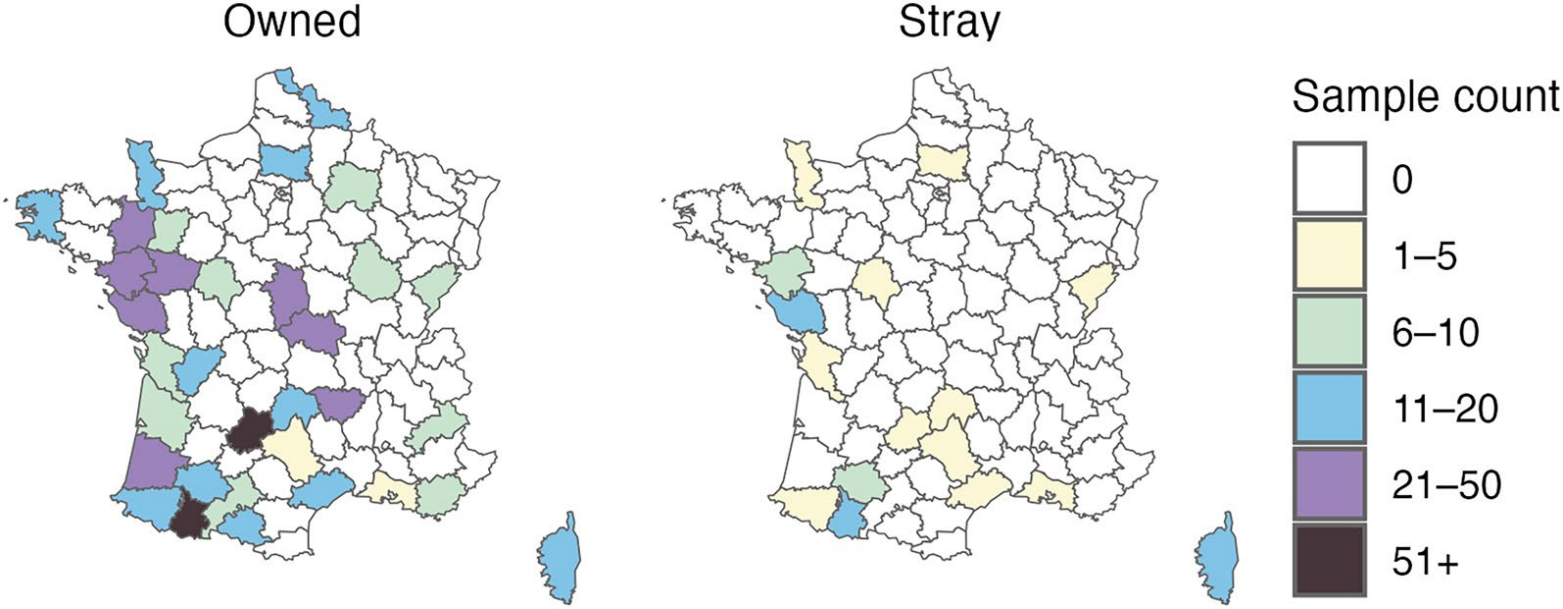
**Geographic distribution of sampling effort by ownership status.** Maps display the number of sampled cats, per department, for owned (left) and stray (right) cats. Owned cats were sampled across 33 departments, with the majority of departments obtaining 6–20 samples (*n* = 21). Samples from stray cats were obtained in 16 departments, with the majority contributing fewer than 6 samples (*n* = 11).

Sampling effort varied by department and ownership status (Figure 1). Owned cats were sampled across 33 departments, with a median of 12 samples (range: 5–98) per department. Conversely, only 16 of those departments also yielded samples from stray cats, with a median of 4 samples (range: 1—16) per department.

### National FIV prevalence

**Figure 2:**
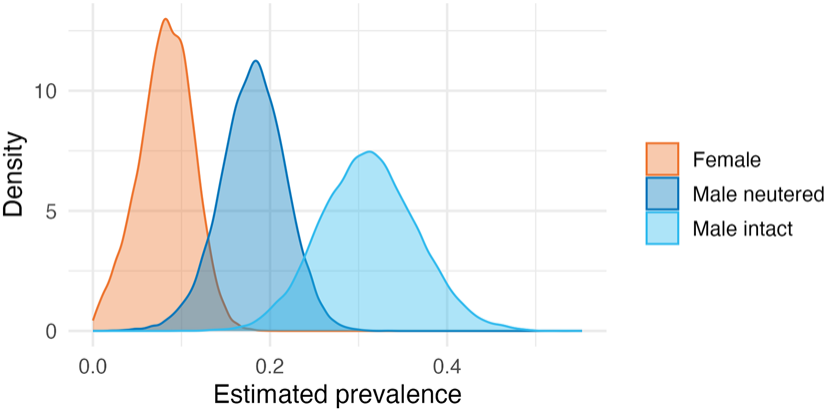
**Posterior density distributions of true FIV prevalence among domestic cats in metropolitan France.** The median estimated prevalence of FIV was 8.4% (95% credible interval: 1.9–13.8%) among females, 18% (11–25%) among neutered males and 31% (21–42%) among intact males. The presence of true differences existing between groups was well-supported, with a 98% or greater probability that prevalence is higher in intact males than both neutered males (by 13% [0.7%–26%]) and females (by 23% [11%–35%]), and is higher among neutered males than females (by 9.8% [0.5%–19%]).

Seropositivity was 18.4% (134/728). The overall true national prevalence of FIV in outdoor cats in France was estimated at a median of 16% (95% credible interval [CrI]: 8.4–20%). When partitioned by ownership status, prevalence was computed at 15% (7.7–20%) among owned cats and 24% (13–36%) among strays; however, the estimated difference between groups did not exclude zero (mean 9.7%, CrI –2.6% to 23%), indicating the existence of a probable albeit less-certain difference between groups. When examined by sex-neuter status, the highest prevalence was observed among intact male cats with a median of 31% (21–42%), followed by neutered males (18%, 11–25%) and then females (8.4%, 1.9–13.8%) (Figure 2). These values were akin to those obtained through Rogan–Gladen estimation (intact males 31% (95% confidence interval [CI]: 22–41%), neutered males 19% (14–24%), and females 9% (5.5–13%)). Posterior comparisons confirmed the high likelihood of the estimated differences (Additional file 2).

### Predictors of exposure

**Figure 3:**
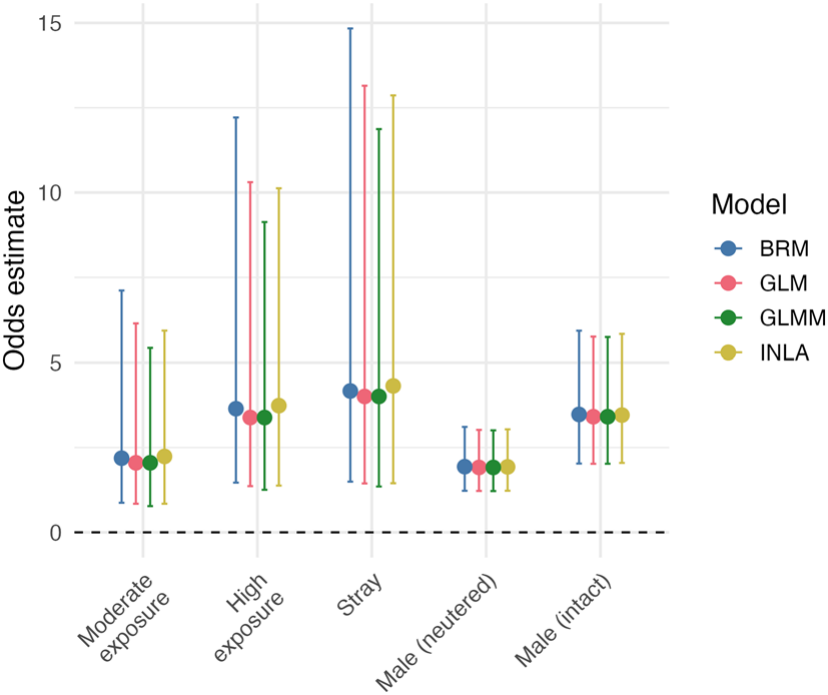
**Posterior mean odds and 95% credible or confidence intervals of FIV seropositivity by predictor.** Model classes consist of a logistic regression model with fixed effects (GLM), a generalized linear mixed model with department-of-origin as a random intercept (GLMM), a spatial model with a BYM2 structured random effect for department adjacency (INLA) and a fully Bayesian GLMM with weakly informative priors (BRM). Female cats with low outdoor exposure (< 1 h outdoors per day) served as the reference category. Cats with moderate exposure (1–12 h outdoors) had twice the odds of being FIV seropositive, cats with high exposure (> 12 h outdoors) had 3–4 times the odds and stray cats had approximately 4–5 times odds. Male neutered and male intact cats had approximately 2 and 3.5 times odds of being seropositive, respectively. Exact values provided in supplementary material (Additional file 2).

Across all models, the odds of FIV seropositivity increased with level of outdoor exposure and was highest among stray cats (Figure 3). Compared with female cats with low outdoor exposure (reference group, with low exposure defined as owned cats never outdoors or outdoors for less than 1 h per day), those with moderate outdoor exposure (owned cats reported outside for more than 1 h but less than 12 h) had approximately twice the odds of being seropositive, those with high exposure (owned cats outside for more than 12 h) had three to four times the odds, and stray cats– presumed always outside and having the strongest association – had odds estimates between 4.0 and 4.3 times higher (Additional file 3). In addition to outdoor exposure, sex and neuter status were also strong predictors of FIV, with neutered male cats having almost twice the odds of being seropositive compared with female cats, and intact male cats having over three times the odds. Despite the observed variability in uncertainty between models, all models yielded consistent effects sizes, demonstrating a strong positive association between FIV seroprevalence, outdoor exposure and the male sex.

### Predicting positivity by age among strays

To identify the best-fitting model that would be used to predict FIV seropositivity by age among strays, we compared the performance of the four models across their frequentist and Bayesian frameworks. Among the frequentist models, the baseline GLM model had the lowest AIC (671.7) and outperformed the GLMM (AIC: 673.7). Among the Bayesian models, the INLA model (WAIC: 672.5) outperformed the BRM (WAIC: 673.5). Given the sparse department-level sample size of stray cats that hindered the stability of spatial effects, the more parsimonious non-spatial GLM was selected to model seropositivity by age among strays. Inclusion of sex as a predictor did not improve the fit (likelihood ratio test:* p* = 0.39), and was therefore excluded from the final prediction model.

**Figure 4:**
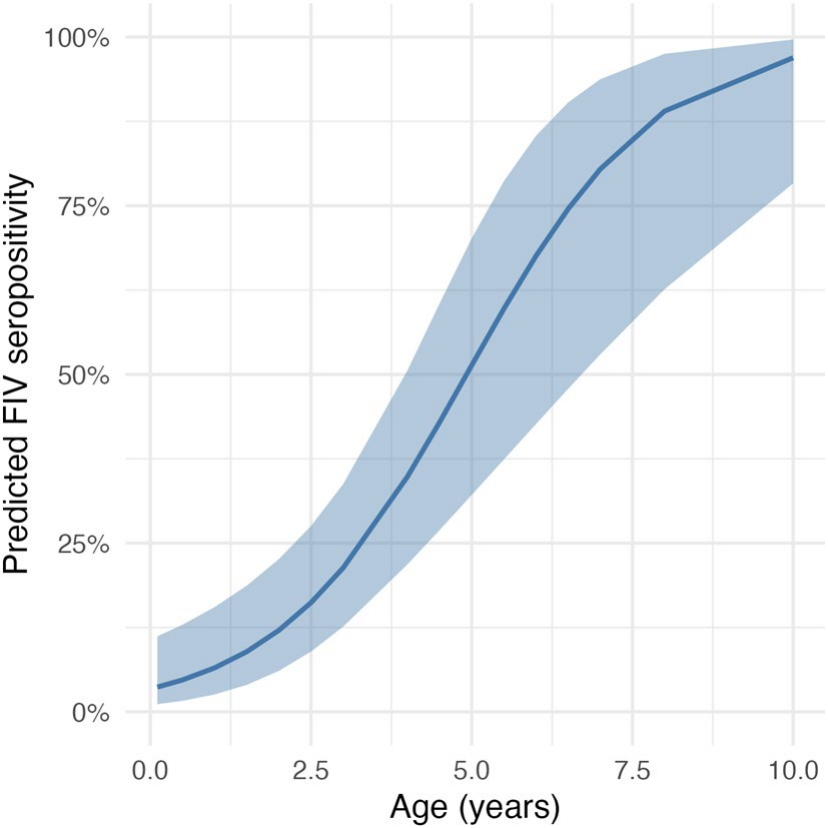
**Predicted probability of FIV seropositivity by age among stray cats.** The curve shows the predicted probability estimates from a logistic regression model using age as a continuous predictor. The shaded region represents the 95% confidence band. The steepest increase in seropositivity probability is seen to occur in the first 5 years, with a median seropositivity probability of 51% (95% confidence interval: 32–70%) by age 5.

Each additional year of age was associated with a doubling of the odds of FIV seropositivity (odds ratio 1.97, 95% CI 1.49–2.81,* p* < 0.001), indicative of a strong age-related increase in risk of infection. Predicted probabilities showed the greatest increase in infection risk occurring during early adulthood, with over a 50% chance of a stray cat being infected by 5 years of age and approaching certainty by age 10 (Figure 4).

### Phylogenetic analysis

**Figure 5:**
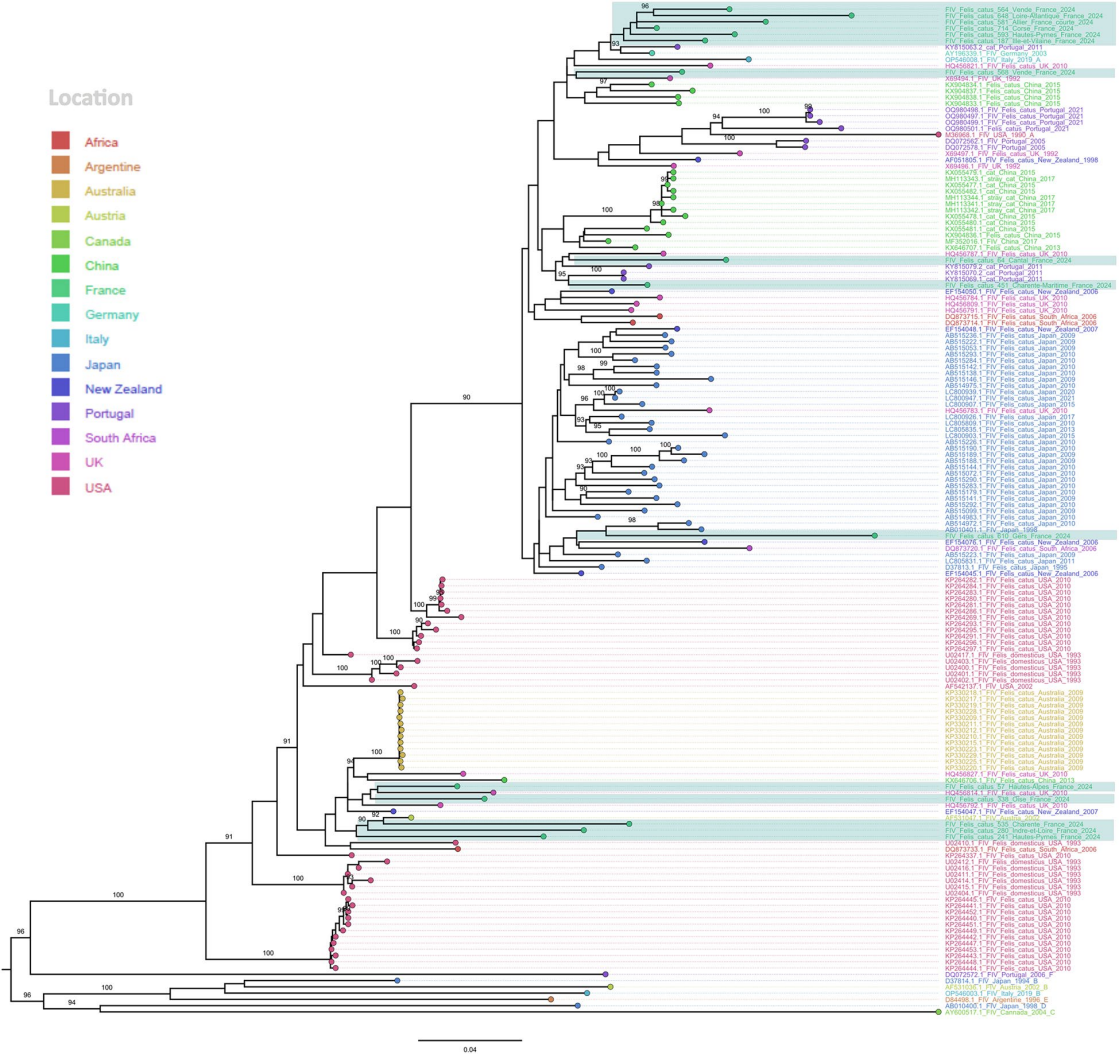
**Maximum likelihood phylogenetic tree.** Sequences generated in this study are highlighted in green. Bootstrap support values > 90 are shown at branches. Scale bar: number of nucleotide substitution per site.

According to the phylogenetic analysis, all FIV strains detected in our study belonged to subtype A. However, based on the topology of the phylogenetic tree, the observed genetic variability of the circulating viruses suggests at least two distinct introductions into France, involving the circulation of viruses with different origins (Figure 5). Although all strains appear to be related to European strains (from Portugal, Italy and Germany), some have different common ancestors supported by strong ulftrafast bootstrap values (> 90). For the strains from Cantal and Charente–Maritime, the bootstrap values provide stronger confidence in identifying their closest relatives, which appear to be Portuguese viruses detected in 2011. Some strains from Charente and Indre et Loire were also strongly related to Austrian viruses detected in 2002. The long branches observed on the phylogenetic tree among the French strains do not suggest a recent introduction, and the closest identified strains date back to 2002 or 2011. These findings highlight the lack of FIV surveillance activities, which is reflected in the limited amount of publicly available data necessary to better understand the international circulation of this virus.

### Metagenomics

To enrich the limited genomic databases currently available for FIV and to support future molecular epidemiology studies, we deep-sequenced one sample (selecting the one with the highest viral load) using Oxford Nanopore Technology. A total of 1.6 million reads were generated from the cat serum sample, with an estimated N_50_ value of 438 bp. Following the filtration step, 166 205 reads, totaling 100.6 million bases, were retained for metagenomic analysis. Taxonomic classification using Kraken2 identified 160 851 reads as being of viral origin. Among these, 100,454 sequences (60.44%) were affiliated with FIV. In addition, 52 595 reads were assigned to Gammaretroviruses, including 34 473 specifically classified as feline leukemia virus (FeLV). Together, these two viral taxa represented 92.08% of the total viral reads. The remaining 13 560 reads were affiliated with various viral species commonly detected in metagenomic studies, but not known to be pathogenic (Additional file 4). A consensus sequence was generated from the FIV-specific reads using the iVar tool. The resulting sequence was 9083 bp in length, with an average read depth of 6664. The consensus sequence has been deposited in the GenBank database under accession number PV976746.

## Discussion

This study provides updated estimates of FIV seroprevalence in France, based on a large and geographically diverse cohort of outdoor-access cats. Using a commercial ELISA, we estimated the median overall national prevalence at 16%. Extrapolated to the estimated 16.6 million cats in France [31], this suggests that over 2.6 million French cats may be infected with FIV. Our findings are in line with a recent literature review [32], which also noted that FIV prevalence is substantially lower in North America yet markedly higher in certain European countries. However, prevalence can vary greatly depending on geographic location and over time, reflecting differences in study design, diagnostic methods and the populations sampled.

The identification of closely related viral sequences from Portugal, Italy and Germany highlights regional differences in viral ancestry and points to complex patterns of virus movement across European borders. These findings underscore the transboundary nature of FIV transmission and the likely role of undocumented cat movement, either through informal adoption, relocation or stray migration. The limited availability of high-quality FIV sequences in public databases currently hampers more detailed phylogeographic inference, emphasizing the need for improved genomic surveillance – all the more so as antibodies directed against certain variants may escape detection by ELISA or POC tests [33]. The successful recovery of over 100 000 FIV-specific reads from a metagenomic Oxford Nanopore dataset demonstrates the utility of untargeted sequencing approaches for full-genome characterization in field samples. To our knowledge, this is the first complete FIV genome sequenced in France in nearly two decades, marking a significant step forward in the molecular characterization of circulating strains. The low abundance of host-derived reads and the absence of bacterial contamination in the metagenomic dataset highlight serum as an optimal matrix for untargeted lentivirus genome sequencing.

Despite the strengths of the dataset (nationally distributed, large sample population), several factors may have affected both the prevalence estimates and predictor associations. The interpretation of sex-neuter-specific prevalence estimates was complicated by the strong association between ownership and neuter status. Subgroups could not be fully stratified owing to sparse representation, and a significant association between ownership and neuter status (*χ*^2^ = 65.3, *p*-value < 0.001) suggested that conditioning on both simultaneously would have introduced collider bias. Selection bias may have also played a role among the stray component of our sample, as stray cats often present to clinics or shelters only under specific circumstances of injury or illness. In-turn, survivorship bias could have resulted in the overestimation of FIV seroprevalence in this group. Exposure misclassification represents an additional source of potential error, as it is subject to owner recall and fails to capture rare but high-risk exposure events such as unobserved escapes. Lastly, spatial heterogeneity associated with urbanization was not accounted for, despite the propensity for strays in denser urban areas to experience higher contact rates and increased FIV transmission risk than those cats in rural settings.

FIV-infected cats can eventually become immunocompromised, which may increase their susceptibility to secondary infections. This immunodeficiency could potentially elevate the risk of developing diseases, such as feline infectious peritonitis [34, 35], or of acquiring and transmitting zoonotic viruses, such as avian influenza viruses and coronaviruses, which have the capacity to infect felids [12, 36, 37]. However, of the 19 H5Nx seropositive cats in this cohort, only three were also FIV seropositive [12].

Importantly, while FIV causes a lifelong infection, most infected cats can live for many years without clinical disease. The idea that a positive test result warrants euthanasia is outdated and unsupported by current evidence. In fact, many infected cats have a lifespan comparable to that of their uninfected counterparts [38], though a subset may eventually develop clinical immunodeficiency of unknown pathogenesis. Therapeutic options for FIV remain limited and controversial. Although some antiviral therapies, such as interferon-based treatments or drugs initially developed for human immunodeficiency virus (HIV), have been proposed, clinical data supporting their use are sparse and inconclusive [6]. No standardized treatment protocols have been universally adopted. A vaccine against FIV is currently licensed in Australia, New Zealand and Japan; however, field studies have shown highly variable efficacy, and the vaccine remains unavailable or unused in most European countries [11, 39, 40]. Given the chronic nature of the infection and the lack of curative or highly effective preventive tools, reducing FIV incidence must rely primarily on behavioural and management interventions. Neutering plays a key role by reducing roaming and fighting behaviour, particularly in male cats [41]. In addition, limiting outdoor access is one of the most effective strategies to prevent exposure, though it may be difficult to implement broadly in populations accustomed to free-roaming lifestyles.

In conclusion, our findings indicate that FIV is well-established in the French cat population and is of particular concern among high-risk subgroups, such as intact males and free-roaming cats. In addition, this study highlights the need for more systematic surveillance, better control strategies focused on behavioural risk reduction, and further research into the pathogenesis, treatment and molecular epidemiology of FIV. These results may help guide veterinary practitioners in making informed decisions regarding FIV prevention, diagnosis and the long-term management of infected cats.

## Supplementary Information

**Additional file 1.** **Sample population.** Descriptive characteristics of sample population by sex, neuter status, ownership, and FIV status.

**Additional file 2.** **Comparison of posterior prevalence estimates between groups.** Differences between male and female, by taking into account the neutered status.

**Additional file 3.** **Estimated odds with 95% confidenceor credibleintervals of FIV seropositivity compared to female cats with low outdoors exposure, by model type and predictor.** Evaluated models consisted of a logistic regression model with fixed effects, a generalized linear mixed model with department-of-origin as a random intercept, a BYM2 spatial model, and a fully Bayesian GLMM with weakly informative priors.

**Additional file 4.** **Metagenomic results.** FIV genome coverage depthand Sankey visualization.

**Additional file 5.** **Information on animals included in the study.** Animals were presented to their veterinarian for a variety of reasons unrelated to the study, ranging from a preventive medicine consultation to surgery.

## Data Availability

The datasets used and/or analysed during the current study are available in Additional file 5. All scripts used for analysis are available at: https://gitlab.envt.fr/epidesa/fivseroprevalencefrance.
