# Supplementary material for: Seroprevalence and genetic diversity of feline immunodeficiency virus in outdoor cats in France
Source: Vet Res. 2025 Dec 4;57:6. doi: 10.1186/s13567-025-01672-z (PMC12781788; doi:10.1186/s13567-025-01672-z)
Supplement: Supplementary file 1 — Additional file 1. Sample population. Descriptive characteristics of sample population by sex, neuter status, ownership, and FIV status. [file 13567_2025_1672_MOESM1_ESM.docx]

**Table S1.** Descriptive characteristics of sample population by sex, neuter status, ownership, and FIV status.

|  | Female cats | | Male cats | |
| --- | --- | --- | --- | --- |
|  | FIV− | FIV+ | FIV− | FIV+ |
|  | Owned | | | |
| Intact | 75 (86.2%) | 12 (13.8%) | 61 (70.9%) | 25 (29.1%) |
| Neutered | 191 (88.8%) | 24 (11.2%) | 203 (79.9%) | 51 (20.1%) |
|  | Stray | | | |
| Intact | 27 (93.1%) | 2 (6.9%) | 19 (59.4%) | 13 (40.6%) |
| Neutered | 10 (76.9%) | 3 (23.1%) | 8 (66.7%) | 4 (33.3%) |
