# Supplementary material for: Seroprevalence and genetic diversity of feline immunodeficiency virus in outdoor cats in France
Source: Vet Res. 2025 Dec 4;57:6. doi: 10.1186/s13567-025-01672-z (PMC12781788; doi:10.1186/s13567-025-01672-z)
Supplement: Supplementary file 2 — Additional file 2. Comparison of posterior prevalence estimates between groups. Differences between male and female, by taking into account the neutered status. [file 13567_2025_1672_MOESM2_ESM.docx]

**Table S2.** Comparison of posterior prevalence estimates between groups

| Group 1 | Group 2 | Difference in mean prevalence (95% CrI) | P (Group 1 > Group 2) |
| --- | --- | --- | --- |
| Intact male | Neutered male | 13.1% (0.7% – 26.2%) | 98.1% |
| Intact male | Female | 22.9% (11.0% – 35.2%) | >99.9% |
| Neutered male | Female | 9.8% (0.5% – 19.1%) | 98.0% |
