# Supplementary material for: Seroprevalence and genetic diversity of feline immunodeficiency virus in outdoor cats in France
Source: Vet Res. 2025 Dec 4;57:6. doi: 10.1186/s13567-025-01672-z (PMC12781788; doi:10.1186/s13567-025-01672-z)
Supplement: Supplementary file 3 — Additional file 3. Estimated odds with 95% confidenceor credibleintervals of FIV seropositivity compared to female cats with low outdoors exposure, by model type and predictor. Evaluated models consisted of a logistic regression model with fixed effects, a generalized linear mixed model with department-of-origin as a random intercept, a BYM2 spatial model, and a fully Bayesian GLMM with weakly informative priors. [file 13567_2025_1672_MOESM3_ESM.docx]

**Table S3.** Estimated odds with 95% confidence (GLM, GLMM) or credible (INLA, BRM) intervals of FIV seropositivity compared to female cats with low outdoors exposure, by model type and predictor. Evaluated models consisted of a logistic regression model with fixed effects (GLM), a generalized linear mixed model with department-of-origin as a random intercept (GLMM), a BYM2 spatial model (INLA), and a fully Bayesian GLMM with weakly informative priors (BRM).

|  | GLM | GLMM | INLA | BRM |
| --- | --- | --- | --- | --- |
| Moderate exposure | 2.05 (0.84 – 6.14) | 2.05 (0.77 – 5.42) | 2.24 (0.84 – 5.93) | 2.22 (0.87 – 6.87) |
| High exposure | 3.38 (1.36 – 10.31) | 3.38 (1.25 – 9.13) | 3.73 (1.38 – 10.13) | 3.73 (1.40 – 11.94) |
| Stray | 4.00 (1.44 – 13.15) | 4.00 (1.35 – 11.87) | 4.32 (1.45 – 12.86) | 4.29 (1.49 – 14.51) |
| Male (neutered) | 1.91 (1.22 – 3.02) | 1.91 (1.22 – 3.01) | 1.93 (1.23 – 3.04) | 1.95 (1.22 – 3.08) |
| Male (intact) | 3.41 (2.02 – 5.76) | 3.41 (2.02 – 5.74) | 3.46 (2.05 – 5.84) | 3.48 (2.08 – 5.94) |
