# Supplementary figures and images for: Seroprevalence and genetic diversity of feline immunodeficiency virus in outdoor cats in France
Source: Vet Res. 2025 Dec 4;57:6. doi: 10.1186/s13567-025-01672-z (PMC12781788; doi:10.1186/s13567-025-01672-z)

**
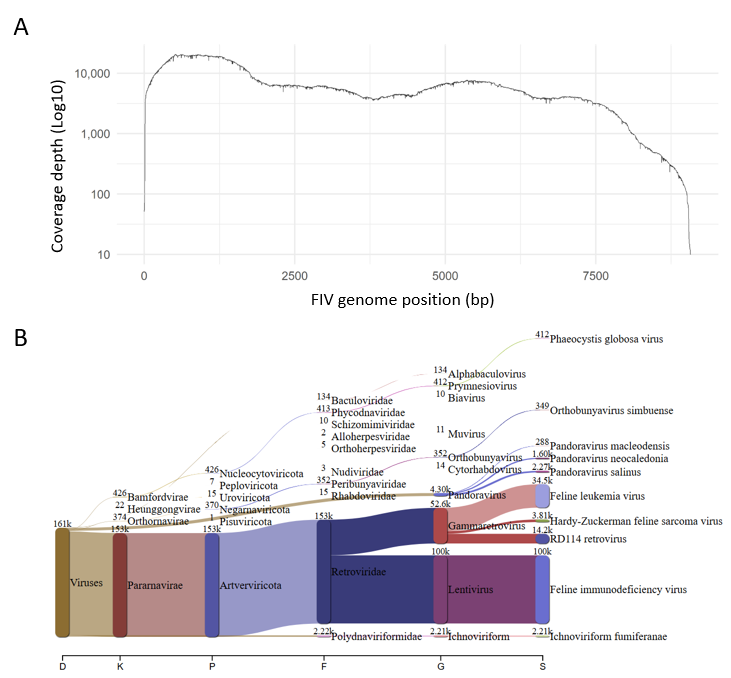
**

**Figure S1: Metagenomic results.** FIV genome coverage depth (A) and Sankey visualization (B).

Supplement: Supplementary file 4 — Additional file 4. Metagenomic results. FIV genome coverage depthand Sankey visualization. [file 13567_2025_1672_MOESM4_ESM.docx]
